# Self-efficacy intervention on health literacy and quality of life in menopausal women of suburban areas

**DOI:** 10.1038/s41598-025-09347-7

**Published:** 2025-07-03

**Authors:** Mansooreh Khandehroo, Nooshin Peyman, Mahdi Gholian-Aval, Hadi Tehrani

**Affiliations:** 1 Student Research Committee, Mashhad University of Medical Sciences, Mashhad, Iran; 2 Department of Health Education and Health Promotion, School of Health, Mashhad University of Medical Sciences, Mashhad, Iran; 3 Social Determinants of Health Research Center, Mashhad University of Medical Sciences, Mashhad, Iran; 4 Department of Health Education and Health Promotion, Faculty of Health, Mashhad University of Medical Sciences, Mashhad, Iran

**Keywords:** Health literacy, Self-efficacy, Quality of life, Menopausal women, Suburban, Quality of life, Population screening

## Abstract

Menopausal women in suburban regions often face challenges in accessing healthcare resources, leading to lower health literacy and reduced quality of life. The aim of this study was to determine the effect of an education intervention based on self-efficacy theory on the quality of life, self-efficacy and health literacy of menopausal women living in suburban areas. This Quasi- experimental study was conducted on 214 suburban menopausal women in Mashhad-Iran from 2021 to 2022. At first, women were divided into intervention and control groups by a simple random method. Intervention group received training in four sessions based on the self-efficacy theory. The questionnaires used in this study were TOFLA (Test of Functional Health Literacy in Adults), Scherer’s self-efficacy and MENQOL (Menopausal Quality of Life). Questionnaires were completed before intervention, immediately, and 3 months later. The mean score of quality of life in the experimental group before intervention was 80 ± 27.33, immediately after intervention was 62.66 ± 22.45, and after 3 months was 51.12 ± 23.57 and this improvement was significant (*p* < 0.001). However, in the control group, there was no significant difference in mean score of quality of life before, immediately and 3 months after the intervention(*p* > 0.05). The mean score of health literacy in the experimental group before the intervention was 9.36 ± 11.36, immediately after the intervention was 20.65 ± 11.41, and three months after the intervention was 21.09 ± 11.78 (*p* < 0.001). Results from the study indicated that educational interventions based on the self-efficacy model is an appropriate strategy to promote quality of life and health literacy in menopausal women.

## Introduction

Menopause one of the important periods in women’s life. Women expend one- third of their life past menopause. women’s knowledge of the changes that occur during middle age and understanding of the reasons for these changes can make it easier to for them to navigate this stage of life. Having Knowledge about health and healthcare can have a positive impact on individuals’ health and well-being. The World Health Organization(WHO) defines quality of life as an individual’s perception of their position in life in the context of the culture and value system in which they live, and obtain about their goals, expectations, communications, and needs. Therefore, all women must live that healthy, and be active in the social and economic the future^1^.

During menopause, women experience physical and psychological changes that can impact their quality of life and interpersonal relationships. one of the problems that women may experience during menopause is Sexual dysfunction which can affect mental health, quality of life, and interpersonal relationship^2^. One of the complications of menopause is depression, which can double the problems of this period and can affected quality of life of women^3^. Anxiety is a condition that can affect quality of life and health of postmenopausal women^4^. The quality of life of postmenopausal women can also be affected by Genitourinary syndrome^5^. Genitourinary syndrome of menopause is a persistent and gradually worsening condition that affects the genital and urinary tissues, largely resulting from reduced estrogen during and post-menopause^6^.

Confidence in one’s abilities is an important and influential factor in the quality of life and controlling middle age symptoms. People with strong self-efficacy believe that they can do any difficult task which can lead to success and hopefulness in work and life. They see difficult tasks as challenges and struggles to become proficient rather than threats that they must avoid. Efficient people in the failure continue to strive that succeed^7^.

Deficiency of health literacy can be a factor that affects the quality of life of individuals, and it is considered a global threat today. Health education aims to raise the level of health literacy which helps individuals make informed decisions about their health and the health of their community^8^. In the study of Rachel Yolizmatores, a clear correlation was seen between health literacy, self-efficacy, knowledge. Quality of life improves with proper and timely education accordance with the needs^9^. This study aimed to determine the effect of educational intervention based on self-efficacy model on promoting quality of life, self-efficacy, and health literacy in menopausal women living in the suburbs of Mashhad, Iran.

## Methods

### Sampling and design

This study was a quasi-experimental with a control group that was conducted in menopausal women aged 40–60 years living in the suburbs of Mashhad (Iran). The study period was from 2020 to 2021. In this study, the elderly was eligible to participate in the study if (a) they had informed consent to participate in the study; (b) Menopausal women with no documented medication use in their health records; (c) they living in the suburbs of the city and speaking Persian. Exclusion criteria were unwillingness to participate in training sessions during the intervention, the absence of more than two sessions in education classes and presence of incurable diseases.

The sample size was estimated based on the information about mean and standard deviation change of behavior scores between two groups, from a similar study^10^, Thus, we calculated the sample size equals to 107 participations in each group, using following formula with consideration α = 0.05, *β* = 0.2, S_1_ = 9.12, S_2_ = 10.07, $\overline{X}$_1_ = 55.85 and $\overline{X}$_2_ = 59.45.

$$n = \frac{{\left( {1.96 + 0.84} \right)^{2} \left( {S_{1}^{2} + S_{2}^{2} } \right)}}{{\left( {\overline{X} _{1} - \overline{X} _{2} } \right)^{2} }}$$

**Fig. 1:**
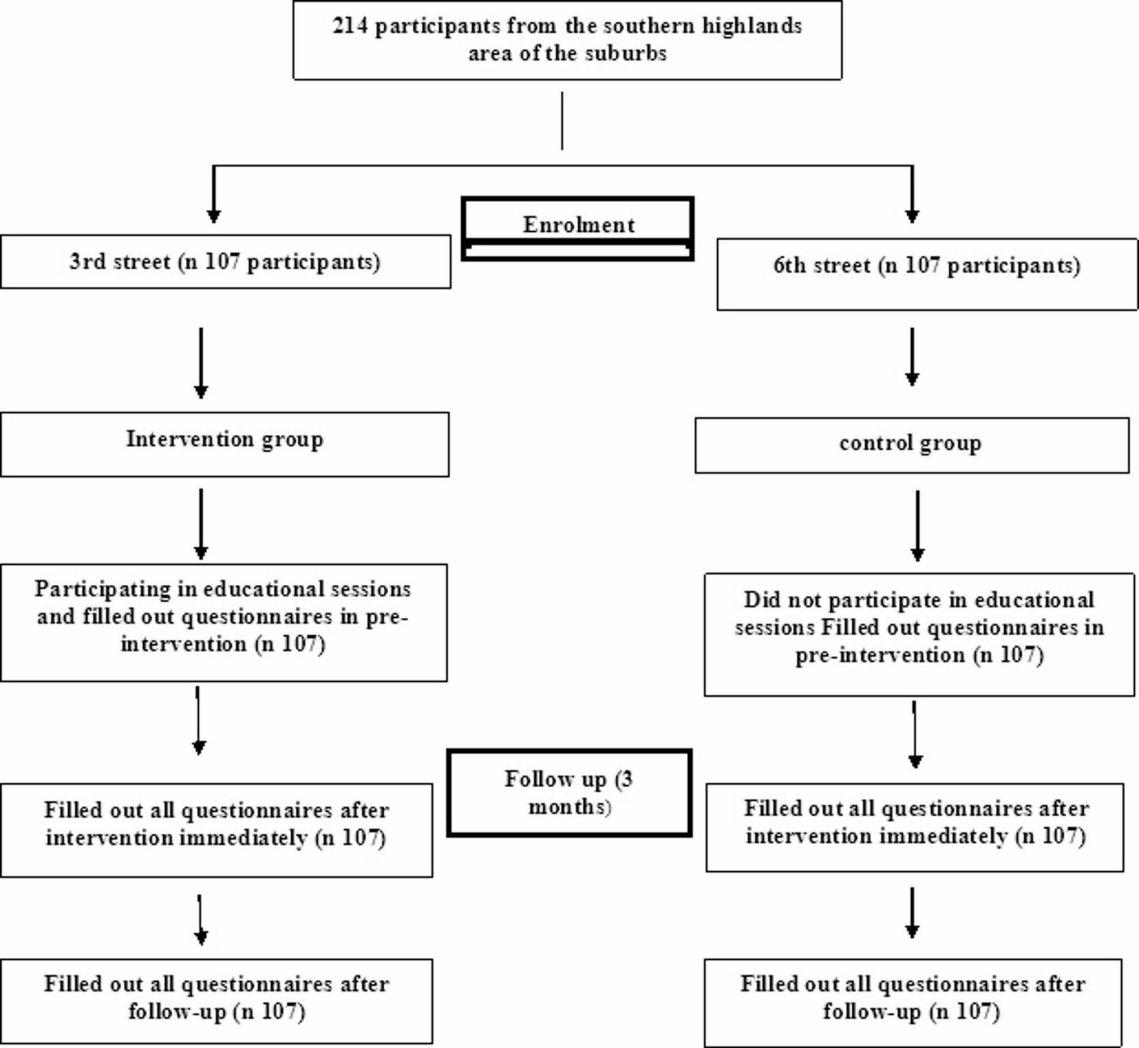
Flow of participants through each stage of the study.

For sampling we used a cluster randomization design, two comparable streets (3 and 6) in Mashhad’s southern marginal zone were selected. Through coin toss randomization, Street 3 became the intervention group while Street 6 served as control. All eligible female residents meeting was enrolled (Fig. 1).

### Tools of assessment

The measurement tools were Short Test of Functional Health Literacy in Adults (S-TOFLA), Menopausal Quality of Life (MENQOL) questionnaire, and sharer self-efficacy questionnaires which previously, its validity and reliability have been confirmed in the study of Nowruzi et al.^11^. The Short Test of Functional Health Literacy in Adults (S-TOFHLA) is a widely recognized instrument for assessing functional health literacy, focusing on reading comprehension and numeracy related to health tasks. This questionnaire contains 36 questions, with each individual’s total score ranging from 0 to 36. In Iran, the full-length Persian version of TOFHLA (which includes the S-TOFHLA components) has been validated and extensively used in health literacy studies among adults. Studies report Cronbach’s alpha coefficients and intraclass correlation coefficients (ICC) for TOFHLA and similar instruments in Iran consistently above 0.7, indicating good reliability^12^.

Scherer’s general self-efficacy questionnaire consists of 17 standard questions, which are graded on a Likert scale from Strongly Disagree (1) to Strongly Agree (5). The maximum score is 85 and the minimum is 17. This questionnaire has been translated and validated by Asgharnejad in Iran^13^. In various Iranian studies, the Cronbach’s alpha coefficient of this questionnaire has been reported to be between 0.75 and 0.89, indicating favorable reliability^14^.

The Menopausal Quality of Life Questionnaire was developed and standardized by John Hildage et al. and consists of 29 questions with psychosocial (7 questions), physical (16 questions), sexual (3 questions), cardiovascular dimension (3 questions), which are graded by Three-option Likert scale. The maximum score is 203 and the minimum is 29. Golrokh Moridi^12^ approved validity and reliability of this questionnaire in Iran. The lower scores obtained in this questionnaire, indicate a better the quality of life.

The questionnaires were completed before intervention, instantly after the intervention, and at 3-months follow-up by participants in both groups. A demographic form was only full filled at baseline.

### Educational intervention

In this study, the intervention was conducted based on self-efficacy model. The intervention was delivered through four 90-minute educational sessions. According to the self-efficacy model, training sessions were conducted based on the progress and success of the participants’ performance in activities to prevent menopausal complications and improve the quality of life during this period. The intervention aimed to realistically design goals and programs and allocate specific rewards for success small goals achieved. The details of the intervention are shown in Table 1. Educational intervention was performed by a specialist health educator at the health care center. Also, review action planning, educational pamphlets, and focus group discussion are used. During the intervention time, participants on the control side had not received any education. They accessed a training package 6 months after the educational intervention.

**Table 1:** Details of the training program.

Sessions	Purpose	Content	Method
First	Raising knowledge’s, attitudes about quality of healthy life All session According to the self-efficacy model: Training sessions were conducted based on the progress and success of the participants’ performance in activities to prevent menopausal complications and improve the quality of life during this period Realistically design goals and programs Allocate specific rewards for success small goals achieved	Healthy lifestyle Dimensions of healthy quality of life and the factors affecting it Ways to improve the quality of life	Lecture Focus group Express experiments
Second	Raising knowledge’s, attitudes about quality of health literacy	Teaching different aspects of health literacy and its importance	Lecture Focus group
Third	Raising knowledge’s, attitudes about self-efficacy	The concept of the importance of self-efficacy Training ways to improve self-efficacy Training the factors affecting self-efficacy	Lecture Focus group Practical relaxation
Forth	Increase performance in the field of self-efficacy and quality of life	Remind, summarize and present successful experiences	Focus group Practical relaxation Present successful experiences

### Statistical analysis

Statistical analysis was performed using SPSS software (version 21). First, the data were checked for normality by Kolmogorov-Smirnov. The characteristics of the participants were described using descriptive statistics. The data were analyzed using independent t-test and Repeated measures. The significance level chosen for this study was *p* < 0.05.

## Results

The Participants were 214 people with a mean age of 53.4 7 ± 7.72. In the control group 53.3%(57) had primary education, 15%(16) middle school, 23.4%(25) had a diploma, and 8.4%(9) had university education. There was no significant difference in education level between the experimental and control groups (*P* > 0.05). In the control group 92.5%(99) were housewives, 5.6%(6) self-employed. 1.9%(2) had government jobs. Occupational status did not differ significantly between the experimental and control groups (*P* > 0.05). In the control group, income status was favorable for 58.9%(63), unfavorable for 27.1%(29), while 14%(15) could not meet basic daily needs. We found no significant between-group differences in income status (*P* > 0.05). Details of the demographic characteristics and matching status of the control and experimental groups were shown in Table 2.

**Table 2:** Frequency and percentage of demographic variables.

Variable		Experimental group		Control group		P-value*
		Frequency	Percentage	Frequency	Percentage	
Occupation	Self employed	2	1.9	6	5.6	> 0.05
	Governmental	2	1.9	2	1.9	
	Housewife	103	96.3	99	92.5	
Income	Favorable	66	61.7	63	58.9	> 0.05
	Unfavorable	28	26.2	29	27.1	
	Inability to meet daily needs	13	12.1	15	14.0	
Education	Primary	61	57.0	57	53.3	> 0.05
	Under diploma	12	11.2	16	15.0	
	Diploma	25	23.4	25	23.4	
	University	9	8.4	9	8.4	

*Chi-square test.

The mean score of quality of life in the experimental group before intervention was 80 ± 27.33, immediately after intervention was 62.66 ± 22.45, (*p* < 0.001), and after 3 months was 51.12 ± 23.57, (*p* < 0.001). This improvement was significant (p-value < 0.001). However, in the control group, there was no significant difference in mean score of quality of life before, immediately and 3 months after the intervention (Table 3).

The mean score of health literacy in the experimental group before the intervention was 9.36 ± 11.36, immediately after the intervention was 20.65 ± 11.41, and three months after the intervention was 21.09 ± 11.78, and this improvement was also significant (p-value < 0.001). However, in the control group, there was no significant difference in mean score of quality of life before, immediately and 3 months after the intervention (Table 3).

The self-efficacy scores in the experimental group were 44.73 ± 13.72, before the intervention, 54.76 ± 14.36, immediately after the intervention, and 55.96 ± 14.80, three months after the intervention. This improvement was also significant (p-value < 0.001). However, in the control group, there was no significant difference in mean score of quality of life before, immediately and 3 months after the intervention (Table 3).

**Table 3:** Mean and standard deviation of variables of quality of life, health literacy and self-efficacy, before, immediately and 3 months after intervention.

Variable		Before intervention		Immediately after intervention		3-month follow-up		p-value*
		Mean	SD	Mean	SD	Mean	SD	
Quality of life	Experimental	80.0	27.33	62.66	22.45	51.12	23.57	< 0.001
	Control	78.92	26.88	78.83	26.76	78.83	26.76	> 0.05
	**p-value	0.79		*p* < 0.05		*p* < 0.001		
Health literacy	Experimental	9.0	11.36	20.65	11.41	21.09	11.78	< 0. 001
	Control	9.30	10.88	9.30	10.88	9.30	10.88	> 0.05
	**p-value	0.12		*p* < 0.05		*p* < 0.05		
Self -efficacy	Experimental	44.73	13.72	54.76	14.36	55.96	14.80	< 0.001
	Control	42.2	15.25	42.36	15.04	42.36	15.04	> 0.05
	**p-value	0.06		*p* < 0.05		*p* < 0.05		

*Repeated measures.

**Independent T-test.

## Discussion

This study aimed to investigate the effect of an educational intervention based on self-efficacy theory on the quality of life, self-efficacy, and health literacy of postmenopausal women who living in the suburban areas. The results generally showed that Self-efficacy training has been found to be effective in improving the health literacy and quality of life of postmenopausal women living in suburban areas. In line with this finding, Hossein Mirzaee Beni in their study conducted on menopausal women showed that self-care education on the health literacy index significantly improves the quality of life of menopausal women and self-care^15^. Another study conducted on 60 menopausal women in Iran also showed that educational intervention based on health literacy is effective in improving the quality of life of menopausal women.

Quality of life refers to an individual’s perception of physical, mental, social, and environmental health state. Various variables such as personal characteristics can have an impact on the quality of life. These variables may include: age, gender, personality and education. organizational factors such as stress experienced in the workplace and the nature of work and tasks can have an impact on the quality of life. Nazarpour’s et al. found that individual and social factors can exacerbate menopausal complications and thus diminished the quality of life^16^. Golyan et al. presented that one of the main factors to improve quality of life is the improvement of lifestyle^17^.

The results of the study suggest that education can significantly increase the average health literacy of women. This finding is consistent with the results of previous studies that attributed a significant improvement in health literacy to health education. A study investigated the effect of educational intervention on health literacy, self-efficacy, and quality of life in women and found that training based on health literacy can improve quality of life^18^. By improving health literacy, women can make informed decisions about their health, engage in self-care practices, and ultimately enhance their overall well-being^19^. In a study conducted by Khanderoo et al. on postmenopausal women, it was shown that education improves women’s health literacy, and increasing health literacy is significantly effective in improving the quality of life^20^. Additionally, a study found that self-care education based on the health literacy index has the potential to improve self-care and quality of life among menopausal women^15^.

The results of the study on self-efficacy showed that after the educational intervention, the mean of self-efficacy in the intervention group increased significantly, while no significant increase was observed in the control group. This suggests that the educational intervention had a positive impact on the self-efficacy of postmenopausal women in intervention group compared to the control group. the results of other studies are similar to the study in question because they also trained the participants about menopause-related self-care1. For example, a randomized controlled trial found that a group-based educational program improved the self-efficacy and self-acceptance of Iranian menopausal women^21^. One study found that self-care education based on self-efficacy theory, individual empowerment model, and their integration improved the quality of life of menopausal women^22^.

### Limitation

In this study, we faced some limitations. First, the self-report questionnaire was used, possibly leading to biases in final outcomes. Second, the presence of Covid-19 and its impact on communication and education of participants, as well as the fear of Covid-19 if they participated in training classes.

## Conclusion

Based on these findings, it can be concluded that education plays a crucial role in increasing the health literacy of women, leading to improved quality of life. These interventions can be particularly beneficial for postmenopausal women living in suburban areas who may face unique challenges in accessing healthcare and health information. Using the results of such studies in the community can have several positive impacts, particularly in promoting women’s health and well-being, making them healthier and more productive throughout their lives, and taking more effective steps toward sustainable development. In addition to providing the necessary knowledge about menopausal symptoms, complications, how to deal with and adapt to them to maintain health and improve the menopausal quality of life, it is important to Create support groups, induce a positive attitude, and promote healthier behaviors among in postmenopausal women.

Educational interventions can also help managers of health services in controlling and reducing the complications of menopause and reducing the resulting burden on the health care system. Authorities can use the results of this study to inform their policies in disease and health services management.

## Data Availability

All data generated or analyzed during this study are included in this published article.
